# A three-dimensional actuated origami-inspired transformable metamaterial with multiple degrees of freedom

**DOI:** 10.1038/ncomms10929

**Published:** 2016-03-11

**Authors:** Johannes T.B. Overvelde, Twan A. de Jong, Yanina Shevchenko, Sergio A. Becerra, George M. Whitesides, James C. Weaver, Chuck Hoberman, Katia Bertoldi

**Affiliations:** 1 John A. Paulson School of Engineering and Applied Sciences, Harvard University, Cambridge, Massachusetts 02138, USA; 2 Department of Chemistry and Chemical Biology, Harvard University, Cambridge, Massachusetts 02138, USA; 3 Wyss Institute for Biologically Inspired Engineering, Harvard University, Cambridge, Massachusetts 02138, USA; 4 Hoberman Associates, New York, New York 10001, USA; 5 Graduate School of Design, Harvard University, Cambridge, Massachusetts 02138, USA; 6 Kavli Institute, Harvard University, Cambridge, Massachusetts 02138, USA

## Abstract

Reconfigurable devices, whose shape can be drastically altered, are central to expandable shelters, deployable space structures, reversible encapsulation systems and medical tools and robots. All these applications require structures whose shape can be actively controlled, both for deployment and to conform to the surrounding environment. While most current reconfigurable designs are application specific, here we present a mechanical metamaterial with tunable shape, volume and stiffness. Our approach exploits a simple modular origami-like design consisting of rigid faces and hinges, which are connected to form a periodic structure consisting of extruded cubes. We show both analytically and experimentally that the transformable metamaterial has three degrees of freedom, which can be actively deformed into numerous specific shapes through embedded actuation. The proposed metamaterial can be used to realize transformable structures with arbitrary architectures, highlighting a robust strategy for the design of reconfigurable devices over a wide range of length scales.

## Abstract (web summary)

Typically, most structures and devices that can be reconfigured are designed with application specific requirements. Inspired by modular origami ideas, Overvelde *et al*. present a mechanical metamaterial enabling the design of three-dimensional structures of arbitrary architecture with tunable shape, volume and stiffness.

## Graphical abstract

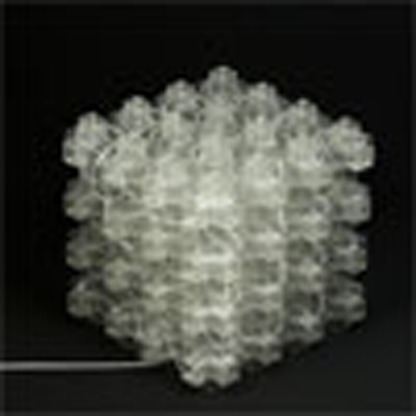

Metamaterials are rapidly appearing at the frontier of science and engineering due to their exotic and unusual properties obtained from their structure rather than their composition. Using origami, the ancient art of paper folding, programmable metamaterials have been previously created from two-dimensional (2D) sheets through folding along pre-defined creases^1^,^2^,^3^,^4^,^5^,^6^,^7^,^8^,^9^,^10^. In particular, origami patterns have proven to be promising in the design of solar panels for space deployment^11^, flexible medical stents^12^, 3D cell-laden microstructures^13^ and flexible electronics^14^. Moreover, it has been shown that cellular metamaterials can be designed by stacking folded layers^4^,^15^,^16^. While almost all proposed origami-inspired mechanical metamaterial designs are based on the Miura-ori fold pattern^11^, which has a single degree of freedom, there are many other origami-like architectures with multiple degrees of freedom that can be used to design highly flexible and deformable 3D structures.

The current work was initially inspired by snapology, a type of modular unit-based origami invented by Heinz Strobl in which paper ribbons are used to create complex geometric extruded polyhedra (Fig. 1a)^17^,^18^. While snapology provides the geometric starting point for our research, our focus here is on the foldability of these structures and how this can lead to new designs for transformable metamaterials. Using this design approach, some of the extruded polyhedral geometries, such as the extruded icosahedron shown in Fig. 1b, are stiff and almost rigid, while others have multiple degrees of freedom and can be easily deformed (see Supplementary Movie 1)^19^. On the basis of these observations, here we focus on an extruded cube^20^ (Fig. 1c) as a fundamental building block and demonstrate both analytically and experimentally that such a structure can be used as a unit cell in the design of foldable reprogrammable matter whose shape, volume and stiffness can be markedly altered in a fully predictable fashion. Moreover, we show that the properties of the resulting 3D mechanical metamaterial can be actively tuned and controlled by strategically placing pneumatic actuators on the edges (hinges) of each unit cell.

## Results

### Characterization of the unit cell

**Figure 2:**
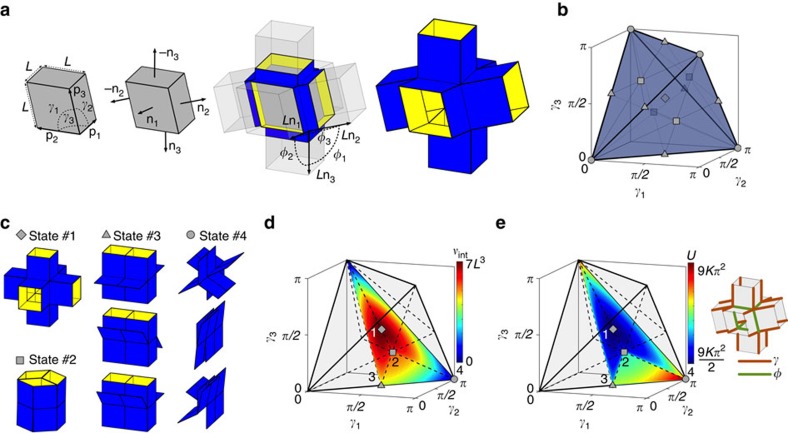
Analysis of the possible shapes of the extruded cube unit cell. (**a**) The shape of the unit cell is found by extruding the edges in the direction normal to the faces of a rhombohedron, and can be fully described by the vectors **p**_1_, **p**_2_ and **p**_3_ spanning the internal rhombohedron. (**b**) Regular tetrahedron containing all combinations of angles (*γ*_1_, *γ*_2_, *γ*_3_) that are attainable. (**c**) State #1, #2, #3 and #4 are configurations that lie in the centre of the regular tetrahedron, on the centre of its faces, on the centre of its edges and on its vertices, respectively. (**d**) Contour plot showing the evolution of internal volume (*v*_int_) of the unit cell as a function of *γ*_1_, *γ*_2_ and *γ*_3_. (**e**) Contour plot showing the evolution of the strain energy (*U*) of the unit cell as a function of *γ*_1_, *γ*_2_ and *γ*_3_. Note that the values of *v*_int_ and *U* are shown on the boundary of a sub-region of the regular tetrahedron, which, because of the symmetry of the unit cell, contains all possible configurations. Moreover, the orange and green lines on the unit cell indicate edges (hinges) whose energy are specified by the *γ* and *φ* angles, respectively.

We construct a unit cell by extruding the edges of a cube in the direction normal to each face (Fig. 2a). This results in a 3D structure with 24 faces connected by 36 edges of length *L*. If, as in the simplified model of rigid origami^21^,^22^, we assume that the faces are rigid and the structure can only fold along the edges, such a unit has three degrees of freedom identified by the angles *γ*_1_, *γ*_2_ and *γ*_3_ (Fig. 2a). Changing these three angles deforms the internal cube into a rhombohedron (a 3D geometry formed from six rhombi) and, more importantly, reconfigures the unit cell into many specific shapes. To describe them, we introduce the vectors **p**_1_, **p**_2_ and **p**_3_, which span the internal rhombohedron (Fig. 2a)


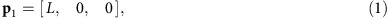



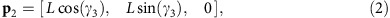



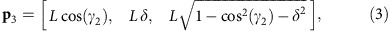


where *δ*=[cos(*γ*_1_)−cos(*γ*_2_) cos(*γ*_3_)]/sin(*γ*_3_). It is important to note that, because of contact occurring between faces, not all combinations of *γ*_1_, *γ*_2_ and *γ*_3_ are possible. All possible combinations of the three angles can be found by requiring the third component of **p**_3_ to be real valued,


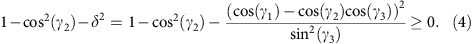


We first rewrite equation (4) as


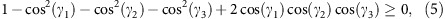


and then solve it for *γ*_3_ to obtain


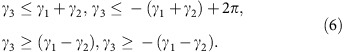


where we have used 0≤*γ*_1_, *γ*_2_, *γ*_3_≤*π*. Note that equation (6) defines a regular tetrahedron with vertices located at (*γ*_1,_ *γ*_2,_ *γ*_3_)=(0, 0, 0), (*π*, *π*, 0), (*π*, 0, *π*) and (0, *π*, *π*) (Fig. 2b). Therefore, only the combinations of angles contained within this domain are attainable.

As shown in Fig. 2c and Supplementary Movie 2, the unit cell can be transformed into multiple highly distinct shapes by varying *γ*_1_, *γ*_2_ and *γ*_3_. At the centre of the tetrahedron (that is, (*γ*_1,_ *γ*_2,_ *γ*_3_)=(*π*/2, *π*/2, *π*/2)) we find the fully expanded configuration (state #1 in Fig. 2c). For this state and for all other combinations of angles.

Inside the tetrahedron, the faces do not come into contact and therefore all three degrees of freedom can be used to deform the unit cell. Alternatively, for configurations that lie on the surface of the tetrahedron, one degree of freedom is constrained due to contact between the faces. As an example, in Fig. 2c we show the configuration that lies at the centre of each face of the tetrahedron (state #2).

When moving to the edges of the tetrahedron, two of the six extruded rhombi flatten, resulting in shapes similar to that of state #3 (Fig. 2c), which lies in the centre of each edge. These edge states still have three degrees of freedom, one associated with shearing of the four open rhombi and two associated with tilting of the two flattened rhombi (Fig. 2c and Supplementary Fig. 1). However, only one degree of freedom is controlled by the *γ* angles, as the edge states correspond to singular points in the *γ* domain. To see this, we focus on the angles *φ*_1_, *φ*_2_ and *φ*_3_ between the vectors normal to the faces of the central rhombohedron (Fig. 2a)


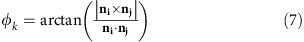



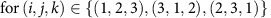
, in which **n**_*l*_ are the normal vectors (Fig. 2a)


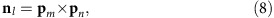



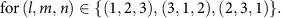


Since for all states on an edge of the tetrahedron two of the vectors spanning the internal rhombohedron are parallel (that is, **p**_*m*_ × **p**_*n*_=0), it follows from equation (8) that one of the normal vectors vanishes (that is, **n**_*l*_=**0**). As a result, when we use equation (7) to evaluate *φ*_*m*_ and *φ*_*n*_, both the numerator and denominator of the argument of the arctangent function are zero, such that both angles are indefinite (and therefore no longer depend on the *γ* angles). Furthermore, by inspecting the deformed unit cell, we find that the two indefinite *φ* angles are related to each other (Supplementary Figs 1 and 4, and Supplementary Table 1), so that each flattened rhombi has one additional degree of freedom, 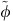, leading to a total of three degrees of freedom (Supplementary Fig. 1).

Finally, at the four vertices of the tetrahedron all three degrees of freedom related to the *γ* angles are constrained, resulting in a state where all six extruded rhombi are folded flat (state #4 in Fig. 2c). For such configuration **p**_1_, **p**_2_ and **p**_3_ are all parallel to each other, so that *φ*_1_, *φ*_2_ and *φ*_3_ cannot be determined as a function of the *γ* angles (Supplementary Fig. 1). Therefore, these vertex states are described by the six angles between the flattened rhombi, 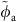, 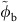, ..., 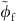 (Supplementary Fig. 1), subjected to the constraint 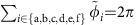, leading to a total of five degrees of freedom.

To quantify the changes in geometry induced by variations in *γ*_1_, *γ*_2_ and *γ*_3_, we calculated the internal volume of the unit cell (that is, the volume enclosed by the 24 faces of the extruded unit), *v*_int_, which is given by


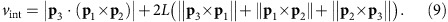


In Fig. 2d we show the values of *v*_int_ on the boundary of a sub-region of the regular tetrahedron, which—because of the symmetry of the unit cell—contains all possible configurations. As expected, the volume is maximum for state #1 (that is, *v*_int_=7*L*^3^) and minimum for state #4 (that is, *v*_int_=0). Moreover, the results indicate that by varying *γ*_1_, *γ*_2_, and *γ*_3_, any intermediate value for the internal volume can be achieved, demonstrating that we have developed a highly effective, yet simple mechanism to markedly alter the shape and volume of the unit cell.

We can also quantify the amount of energy required to deform the unit cell into a given state. By assuming that each of its 36 active edges acts as a linear rotational spring of stiffness *K*, the total strain energy, *U*, can be determined according to


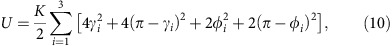


where we assumed that for each edge zero energy is associated to a 180° angle between the two connected faces (so that they form a flat surface) and we have used the fact that 24 edge angles are directly specified by *γ*_1_, *γ*_2_ and *γ*_3_ (orange edges in Fig. 2e) and the remaining 12 edges are defined by equation (7) (green edges in Fig. 2e). While equation (10) can be used to determine the strain energy associated to states lying inside or on the surface of the tetrahedron domain, to characterize the edge states the energy should be modified as


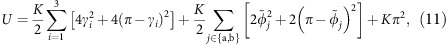



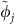
 being the additional degrees of freedom related to the tilting of the flattened rhombi (Supplementary Fig. 1). Similarly, for the vertex states we have


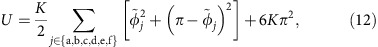


with 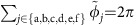.

The results reported in Fig. 2e show that the energy is minimum for state #1 (*U*=9*Kπ*^2^/2). Since this is the only minimum, we expect it to be the preferred configuration when no external force is applied. Additional energy, Δ*U*, needs to be applied to deform the system into any other configuration. For example, to change the unit cell from state #1 to state #2, Δ*U*=11*Kπ*^2^/6, while 3*Kπ*^2^/2≤Δ*U*≤5*Kπ*^2^/2 to reach state #3. Note that a range in Δ*U* exists to deform the unit cell to state #3 and any state on the edges of the tetrahedron, corresponding to different orientations of the flattened extruded rhombi (Fig. 2c and Supplementary Fig. 1). Finally, a fully flattened state (#4) requires an energy of *U*=9*Kπ*^2^, but also in this case different orientations of the flattened extruded rhombi result in an energy range (23*Kπ*^2^/3≤*U*≤10*Kπ*^2^). Most importantly, our analysis indicates that the energies required to deform the unit cell lie within a relatively small range (9*Kπ*^2^/2≤*U*≤10*Kπ*^2^), as the highest energy state is only a factor 20/9 higher then the lowest one. Therefore, we expect that all states can be reached by applying external forces of similar magnitude.

**Figure 3:**
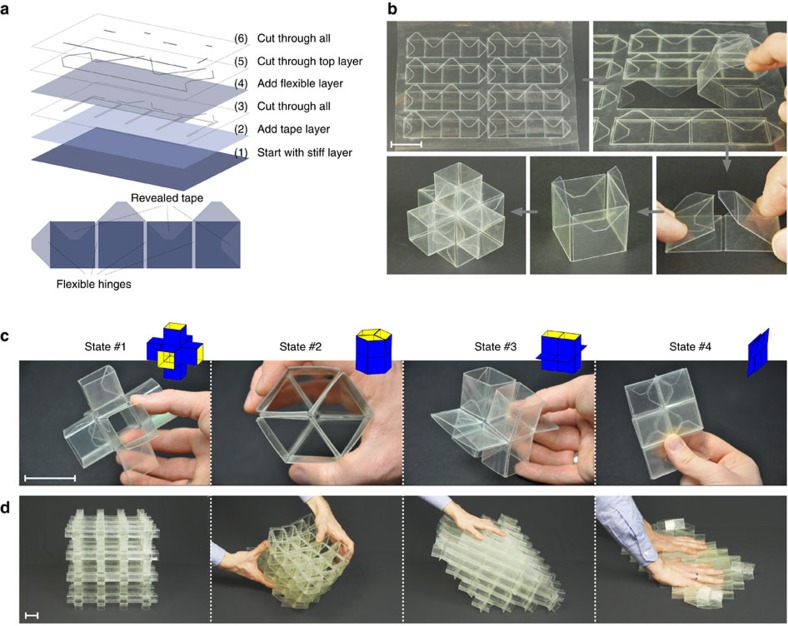
Fabrication and deformation of a single extruded cube unit cell and the corresponding mechanical metamaterial. (**a**) The unit cells were fabricated using three layers: two outer layers of polyethylene terephthalate (with thickness *t*=0.25 mm and 0.05 mm) and a layer of double-sided tape (*t*=0.05 mm) in the middle. The layers were cut in three steps to form flat building blocks with both flexible and rigid regions. (**b**) The extruded rhombi were formed by simply removing the building blocks from the layered sheet, folding them and sticking their ends together using the revealed adhesive tape. To form the unit cell, six cubes were attached together using the double-sided tape incorporated into the layered sheet. (**c**) State #1, #2, #3 and #4 can be realized by simply applying a compressive load. (**d**) A highly flexible mechanical metamaterial with a cubic microstructure was formed by connecting the outer edges of 64 identical unit cells. An external force can trigger a collective behaviour which shapes the cubic crystal into a number of different configurations. Scale bars, 3 cm.

Next, we validated our findings by fabricating and testing a centimeter-scale prototype of the unit cell. To manufacture robust, thin-walled unit cells for which most of the deformation is focused at the edges, we used nearly inextensible polymeric sheets of varying thickness and an efficient stepwise layering and laser-cutting technique (see Fig. 3a,b and the ‘Methods' section)^1^,^5^. In Fig. 3b we show the resulting unit cell; exactly as predicted by our analysis, the structure shapes into the fully expanded state #1 (that is, *γ*_1_=*γ*_2_=*γ*_3_=*π*/2) when no force is applied to it. However, the shape and volume of the unit cell can be altered by manually applying a force, and all configurations predicted by our analysis can be easily realized (Fig. 3c).

### 3D transformable metamaterial

Having identified a highly flexible and deformable 3D unit cell, we next show that it can be used to form a mechanical metamaterial whose shape and volume can be markedly altered. Such metamaterial can be constructed by connecting multiple unit cells through their outer edges. It is important to note that both the number of unit cells and their connections affect the number of degrees of freedom of the assemblies (see Supplementary Fig. 2 for details). In general, we find that large enough connectivity is required for the assembly to be characterized by the same folding modes of the constituent unit cells.

Here, we connected the outer edges of 64 identical unit cells to fabricate a 4 × 4 × 4 cubic crystal (Fig. 3d). The snapshots shown in Fig. 3d (see also Supplementary Movie 3) indicate that the assembly still has three degrees of freedom and deforms in exactly the same manner as each constituent unit cell. Therefore, an external force can trigger a collective behaviour which transforms the mechanical metamaterial into a number of different configurations. In fact, by changing the microstructure (that is, the unit cells) into the various possible configurations described in Fig. 2, the macrostructure of the mechanical metamaterial can be significantly altered and its initial cubic shape deforms either into an extruded hexagon, a rhombohedron, or even a completely flat 2D state (Fig. 3d). Note that for the states inside the regular tetrahedron the total volume occupied by each unit cell, *v*, can be calculated as


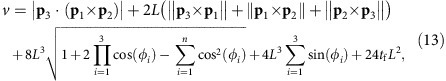


where *t*_f_ is the thickness of the extruded faces of the unit cell. As expected, we find that the total volume is maximum for the fully expanded case (*v*_state#1_=27*L*^3^+24*t*_f_*L*^2^) and it is minimum in the fully flat state (*v*_state#4_=24*t*_f_*L*^2^). Therefore, the maximum volume ratio, 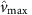, that can be achieved equals


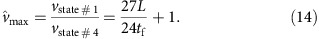


Equation (14) clearly shows that the maximum change in volume depends on the ratio *L*/*t*_f_ and, therefore, on the material and techniques used to fabricate the extruded geometry. Focusing on the structure fabricated in this study, for which *L*=30 mm and *t*_f_=0.35 mm, we have 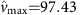. Therefore, approximately a 100-fold volume reduction is achieved when our metamaterial is completely flattened, making the unit cell highly suitable for the design of deployable systems.

### Actuation

**Figure 4:**
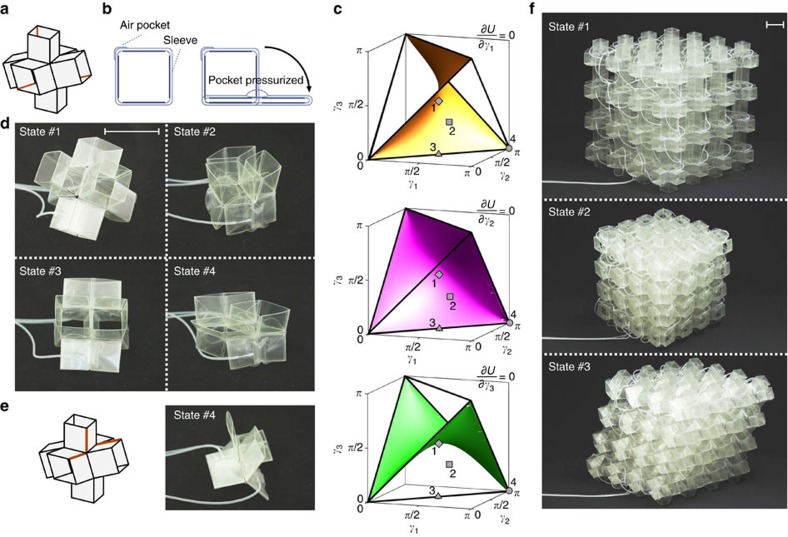
Actuation of the unit cell and the corresponding mechanical metamaterial. (**a**) To freely transform the entire unit cell, inflatable air pockets are placed on the hinges highlighted in orange (see the ‘Methods' section). (**b**) An internal pressure in the air pockets results in a moment in the hinges, causing the extruded rhombus to flatten. (**c**) Surfaces for which ∂*U*/∂*γ*_*i*_=0. When moving between two states connected by a path that remains on one of these surfaces, the corresponding *γ*_*i*_ angle does not have to be actuated. (**d**) Configurations obtained by actuating the unit cell (with 3 actuators). (**e**) Improved actuation strategy to reach state #4. As expected state #4 does not fold completely flat, but instead deforms into the state with lowest strain energy for which *φ*_1_=*φ*_2_=*φ*_3_=2*π*/3. (**f**) Actuation of the mechanical metamaterial (with 96 actuators). Note that all structures are actuated by connecting the air pockets to three separate syringes through transparent tubes. Scale bars, 3 cm.

While so far we have shown that the shape and volume of our metamaterial can be altered by manually applying a force (Fig. 3d), we now explore a possible distributed actuation approach to programme and control its shape. To this end, we position inflatable pockets on three hinges of the unit cells to control the *γ* angles (Fig. 4a). Upon pressurization, these pockets apply a moment to the hinges, flattening them and forcing the extruded rhombi to change their shape (Fig. 4b and Supplementary Movie 4).

To determine which inflatable pockets should be pressurized to move between different configurations, we first note that the total energy, *T*, in our system is given by


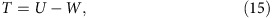


where *W* indicates the work done to actuate the system and *U* is the total strain energy defined in equations (10, 11, 12). The equilibrium configurations can then be found by minimizing *T* with respect to all degrees of freedom, yielding


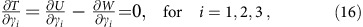


for all the states internal and on the faces of the regular tetrahedron. Since ∂*W*/∂*γ*_*i*_ is the moment applied by the inflatable pocket to the hinge with angle *γ*_*i*_, equation (16) indicates that, in order to move between two states, the actuator controlling the *γ*_*i*_ angle need to be pressurized if


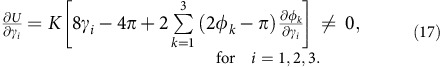


Interestingly, some states inside the regular tetrahedron can be reached without actuation of all three *γ* angles. In fact, we numerically find that ∂*U*/∂*γ*_*i*_=0 defines three surfaces within the regular tetrahedron (Fig. 4c). As a result, when moving between two states connected by a path that remains on one of these three surfaces, no moment needs to be applied to the hinge with angle *γ*_*i*_.

It follows from our analysis that state #2 and any other state on the faces of the tetrahedron can only be reached if all three inflatable pockets are pressurized to different levels (Fig. 4d). This is because it is not possible to connect state #1 to a state on the face of the tetrahedron through a path that remains on one of the surfaces shown in Fig. 4c. In contrast, any of the edge or vertex states can be reached from state #1 while remaining on one of these surfaces, indicating that only two pockets need to be inflated (Fig. 4d). The only exception is the highly symmetric state #3, which only requires pressurization of one actuator since two moments are equal to zero when moving in a straight path between state #1 and #3 (Fig. 4c). We also find that, when trying to reach state #4 by inflating two pockets, the six extruded rhombi do not flatten completely (Fig. 4d), suggesting that the force applied by actuators is insufficient to achieve this configurational change. In fact, all extruded rhombi can be completely flattened by placing one of the actuators on a different hinge, and actuating the three actuators simultaneously (Fig. 4e). Note that the unit cell does not fold completely flat, but instead deforms into the state with the lowest strain energy with 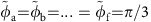, as predicted by equation (12). In order to reach a completely flat configuration, additional air pockets should be placed on some of the hinges highlighted in green in Fig. 2e to control the 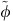 angles.

Having demonstrated that the shape of the unit cell can be controlled by pressurizing embedded inflatable pockets, we now extend this approach to the 4 × 4 × 4 mechanical metamaterial. First, we note that, although the metamaterial still has three degrees of freedom, three air pockets do not provide enough force to simultaneously transform all of its unit cells. Therefore, we distributed 96 air pockets on the outer extruded rhombi of the mechanical metamaterial and actuate each degree of freedom by simultaneously inflating 32 air pockets (Supplementary Movie 5). The snapshots shown in Fig. 4f demonstrate that the shape of our metamaterial can be altered by pressurizing the air pockets. In fact, we could shape all the unit cells of the metamaterial into states #2 and #3. However, the number of actuators was not sufficient to generate high enough forces to achieve state #4.

Finally, we note that since the state domain in Fig. 2b is convex and there is only one energy minima, fully depressurizing the pockets returns the metamaterial to the fully expanded state #1. This means that constant actuation is required to maintain a configuration in a state different from state #1. An interesting approach to mitigate this issue could be to identify different extruded unit cells whose energy is characterized by multiple energy minima, enabling multiple stable configurations. Such multistability, which has already been explored for other origami patterns such as the Miura-ori^6^,^8^, the square twist^9^ and even paper folding bags^23^, might prove useful to further improve actuation and deployment of the proposed structures.

### Load carrying capacity

**Figure 5:**
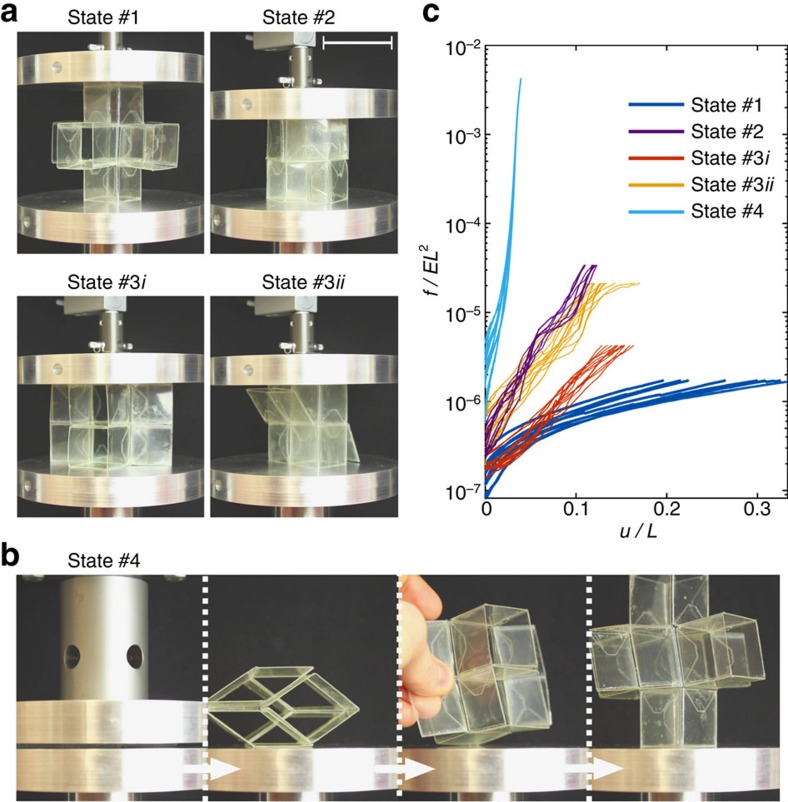
Uniaxial compression of an extruded cube unit cell pre-folded into four different states. (**a**) Snapshots of the loaded unit cell for states #1, #2 and #3. (**b**) The unit cell is folded into state #4 and then compressed by applying 10,000 N. Remarkably, the fully expanded state can be recovered after removal of the load. (**c**) Force-displacement curves under uniaxial compression of a single unit cell at different configurations. Note that the initial force at zero displacement indicates the force required to maintain the unit cell into its pre-deformed (folded) state. Scale bar, 4 cm.

While so far we have focused on the large geometric changes that can be induced in the mechanical metamaterial, such modifications can also be harnessed to alter its mechanical properties. For example, the stiffness of the metamaterial varies significantly for different configurations, as internal contact arises when the unit cells are deformed to configurations that lie on the faces, edges or vertices of the regular tetrahedron (Fig. 2b,c). Such contact constraints further deformation and therefore effectively increases the material's stiffness in certain directions, which can be harnessed to increase the structure's load carrying capacity. Note that the structure can only carry load when the folding motion is fully constraint, and therefore not during a transformation between states. To demonstrate this effect, we performed uniaxial compression tests on a single unit cell shaped into five different configurations (Fig. 5). Since these tests were performed on a single unit cell, they cannot directly be used to predict the response of the metamaterial, but we expect the metamaterial to show qualitatively a similar increase in load carrying capacity, arising from internal contact.

In these tests, the unit cell is first shaped into a specific configuration and placed between the two plates of a uniaxial compression machine (Fig. 5a,b). Then, it is further compressed in vertical direction while ensuring that it remains in the same configuration. In Fig. 5c we report the evolution of the normalized measured force, *f*/(*EL*^2^), as a function of the normalized applied compressive displacement, *u*/*L*, in which *E*=2.6 GPa is the Young's Modulus of PET^24^. The results indicate that for all configurations except for state #1 a non-zero force *f* is measured before applying any compressive displacement (that is, at *u*/*L*=0). This is the force required to shape and maintain the unit cell into the specific configuration and, as expected, is found to be maximum for state #4. Moreover, the experimental data also shows that the increase in force measured as a function of the applied displacement depends on the shape of the unit cell, indicating that the stiffness of the structure is highly affected by both its configuration and the direction of loading. More specifically, the lowest increase in measured force was observed for state #1, since all the deformation is focused at the hinges and no contact occurs. In contrast, the fully flat state #4 showed the largest increase in force upon compression in the direction perpendicular to the flattened rhombi, since it is fully compacted and therefore behaves similar to the bulk material. Note that, although the observed behaviour was not fully elastic, even after applying 10,000 N to the unit cell in state #4 no permanent damage was observed in the hinges, and with some additional manipulations, the fully expanded configuration was recovered after the removal of the applied load (Fig. 5b and Supplementary Movie 6). This suggests that the strains induced by folding the hinges are not large enough to cause any permanent damage such as fracture or creases (see Supplementary Fig. 3 for details). It is expected that the fully expanded configuration completely recovers without additional manipulations when ideal elastic hinges are used. Finally, comparison between the force-displacement response measured for states #3*i* and #3*ii*, indicates that the load carrying capacity of the unit cell depends greatly on the orientation of the unit cells with respect to the direction of loading.

## Discussion

In summary, we have introduced a 3D programmable mechanical metamaterial whose shape, volume and stiffness can be actively controlled, making it ideally suited for the design of deployable and reconfigurable devices and structures. While in this study we focussed on a design based on an extruded cube, many other 3D unit cells with multiple degrees of freedom can be constructed, starting from any convex polyhedra with equal edges and extruding its edges in the direction normal to the faces. Moreover, depending on the characteristic size of the unit cell, different and remote types of actuation can be used to deform the structure, including heat^1^,^25^,^26^,^27^,^28^, swelling^29^ and magnetic fields^30^,^31^. In fact, our choice of using tethered pneumatic actuation was motivated mainly by the fact that such actuators are easy and inexpensive to fabricate and can typically generate reasonable forces^32^.

By exploiting origami's scale-free geometric character, our approach can be extended to the micro- and nano-scale, as well as to the meter-scale. Since the transformation modes described for this material operate independently of the object's macro-scale external geometry, this transformable metamaterial can be machined into any desired architecture. For example, it can be used to design reconfigurable tubular stents with millimeter-scale features that can easily fit through small openings while in their flat state, as well as centimeter-scale foldable chairs and meter-scale deployable domes (Supplementary Movie 7).

Although in this study we have demonstrated the concept at the centimeter-scale, recent developments in micro-scale fabrication and actuation open exciting opportunities for miniaturization of the proposed metamaterial. In fact, origami-inspired metamaterials at the micro-scale could be manufactured by using self assembly^33^ or stress within thin films^34^,^35^,^36^, and by taking advantage of recent developments in hinge construction at small scale for laminate-based mechanisms^12^,^13^,^25^,^26^,^27^,^28^,^34^. This represents a significant advantage for the proposed structures over structures composed of rods connected by rotational joints, which are challenging to fabricate at a very small scale. Therefore, we believe that our approach can result in simplified routes for the design of transformable structures and devices over a wide range of length scales.

## Methods

### Fabrication of the unit cell

The unit cells were fabricated from thin polymeric sheets using an efficient stepwise layering and laser-cutting technique, as shown in Fig. 3a. To fabricate each of the six extruded rhombi that together form a unit cell, we started from a nearly inextensible polyethylene terephthalate sheet with thickness of *t*=0.25 mm, covered with a double-sided tape layer (3 M VHB Adhesive Transfer Tapes F9460PC) with a thickness of *t*=0.05 mm. Cutting slits were introduced into the bilayer using a CO2 laser system (VLS 2.3, Universal Laser Systems), after which a second, thinner polyethylene terephthalate layer (*t*=0.05 mm) was bonded to the tape. A second cutting step with low power was then performed to machine only the top layer. Finally, additional slits were introduced through all three layers. As shown in Fig. 3b, the extruded rhombi could then be formed by removing the parts from the layered sheet, and bonding their ends together. Note that no glue was used in this step, but only the double-sided tape already incorporated into the parts. The unit cell was then formed by attaching together six of the extruded rhombi, again using the double-sided tape incorporated into the parts. For this study, we fabricated unit cells with *L*=30 mm and almost rigid faces with a thickness of *t*_*f*_ =0.35 mm. Moreover, the hinges have a thickness of *t*_h_=0.05 mm and a width of *w*_h_=1.5 mm. Note that we tested multiple values for *t*_f_/*t*_h_, and found that a thickness ratio *t*_f_/*t*_h_=7 provided a good balance between flexibility of the hinges and rigidity of the faces.

### Fabrication of the inflatable actuators

To actuate the unit cells and the metamaterial, we fabricated air pockets and embedded them within sleeves, so that they could be easily applied to the hinges. The air pockets were formed by placing two polyvinyl chloride sheets of thickness *t*=0.075 mm in a hot press and sealing them for 200 s at 175°C. A rectangular piece (14 × 24 mm) of PTFE-Coated Fibreglass Fabric was placed between the polyvinyl chloride sheets during the sealing process and then removed through a small opening, creating the internal pocket. To allow inflation, a small Polyethylene (PE) tube was inserted and glued in the same opening using a Cyanoacrylate based glue. Finally, the units cells were actuated by positioning the sleeves around the extruded rhombi with the air pockets aligned to the hinges, and by inflating the air pockets using syringes attached to the PE tubes.

### Compression tests

We characterized the response of the unit cells under uniaxial compression using a single-axis Instron (model 5544A, Instron, MA, USA). The response of the unit cells configured in states #1 to #3 were tested using a 100 N load cell at a compression rate of 5 mm min^−1^, while state #4 was tested using a 50,000 N load cell at a rate of 0.5 mm min. Each test was repeated nine times and three different unit cells were used. We loaded the samples until the point where debonding between the three layers forming each face started to occur. Furthermore, we ensured that there was enough friction between the sample and the testing machine so that no folding occurred.

## Additional information

**How to cite this article:** Overvelde, J. T. B. *et al*. A three-dimensional actuated origami-inspired transformable metamaterial with multiple degrees of freedom. *Nat. Commun.* 7:10929 doi: 10.1038/ncomms10929 (2016).

## Supplementary Material

Supplementary Information Supplementary Figures 1-4, Supplementary Table 1 and Supplementary References.

Supplementary Movie 1 Stiffness of extruded polyhedral. Our work is inspired by snapology, a type of modular unit-based origami in which paper ribbons are folded and assembled to create complex geometric extruded polyhedra. Interestingly, we found that some of the resulting geometries (such as the extruded icosahedron) are stiff and almost rigid, while others (such as the extruded cube) have multiple degrees of freedom and can be easily deformed.

Supplementary Movie 2 Possible shapes of the unit cell. The unit cell considered in this study can be transformed into multiple highly distinct shapes by varying γ1, γ2 and γ3. Because of contact occurring between its faces, only the combinations of angles contained within the regular tetrahedron with vertices at (γ1, γ2, γ3) = (0, 0, 0), (π, π, 0), (π, 0, π) and (0, π, π) are feasible.

Supplementary Movie 3 Transformable metamaterial. The highly flexible unit cell can be used to form mechanical metamaterials whose shape and volume can be dramatically altered. Here, we connected the outer edges of 64 identical unit cells to form a 4 × 4 × 4 cubic crystal. Importantly, the assembly does not constrain any degrees of freedom, so the mechanical metamaterial deforms in exactly the same manner as each constituent unit cell.

Supplementary Movie 4 Actuation of the unit cell. The shape and volume of the unit cell can be actively programmed by strategically positioning inflatable pockets on the hinges of the unit cell. By pressurizing the air pockets, the shape of the unit cell can be effectively controlled.

Supplementary Movie 5 Actuation of the metamaterial. Similar to the unit cell, the shape and volume of the metamaterial can be actively programmed by strategically positioning inflatable pockets on the hinges of the unit cells.

Supplementary Movie 6 Recovery of the unit cell. The response of the unit cell is always elastic. Even after applying 10,000N no permanent deformation is observed and the fully expanded configuration is recovered after the removal of the applied load.

Supplementary Movie 7 Structures with extreme tunability of shape, volume and stiffness. Our approach can be extended to design deployable and reconfigurable structures and devices over a wide range of length scales. For example, it can be used to design millimeter-scale reconfigurable tubular stents, as well as centimeter-scale foldable chairs and meter-scale deployable domes.

## Figures and Tables

**Figure 1:**
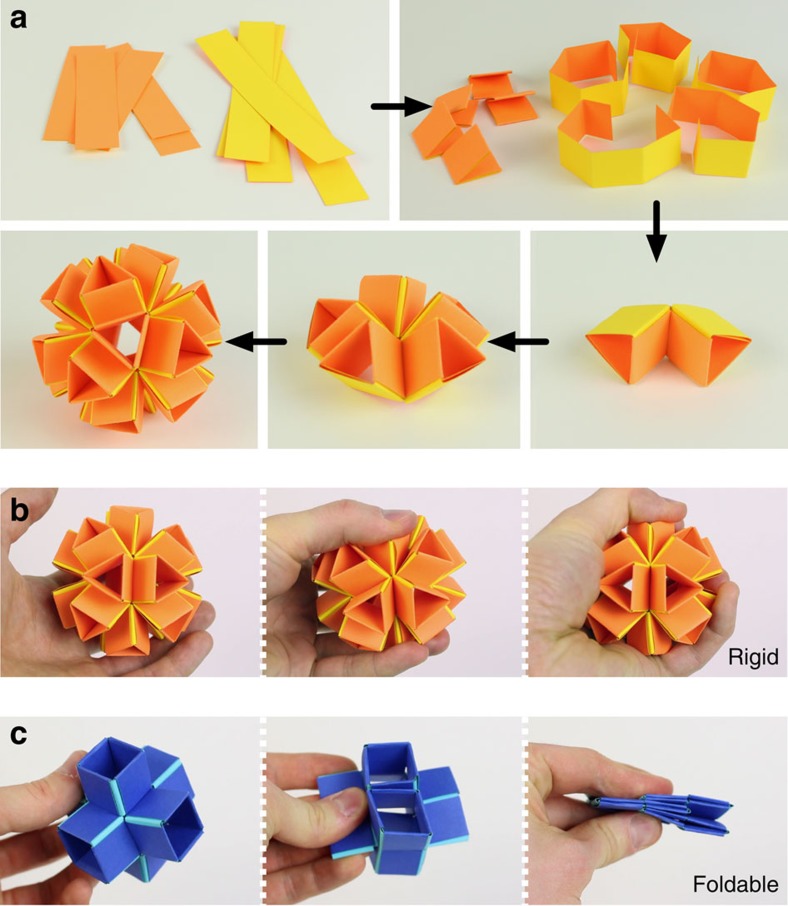
Our work is inspired by snapology. (**a**) Snapology is a type of modular, unit-based origami in which paper ribbons are folded, and ‘snapped' together to assemble extruded polyhedra, such as the extruded icosahedron shown. (**b**) Some of the geometries that can be made in this way, including the extruded icosahedron, are almost rigid. (**c**) In contrast, other geometries, including the extruded cube, have multiple degrees of freedom and can be easily deformed.
