# Supplementary material for: A three-dimensional actuated origami-inspired transformable metamaterial with multiple degrees of freedom
Source: Nat Commun. 2016 Mar 11;7:10929. doi: 10.1038/ncomms10929 (PMC4793042; doi:10.1038/ncomms10929)
Supplement: Supplementary Information — Supplementary Figures 1-4, Supplementary Table 1 and Supplementary References. [file ncomms10929-s1.pdf]

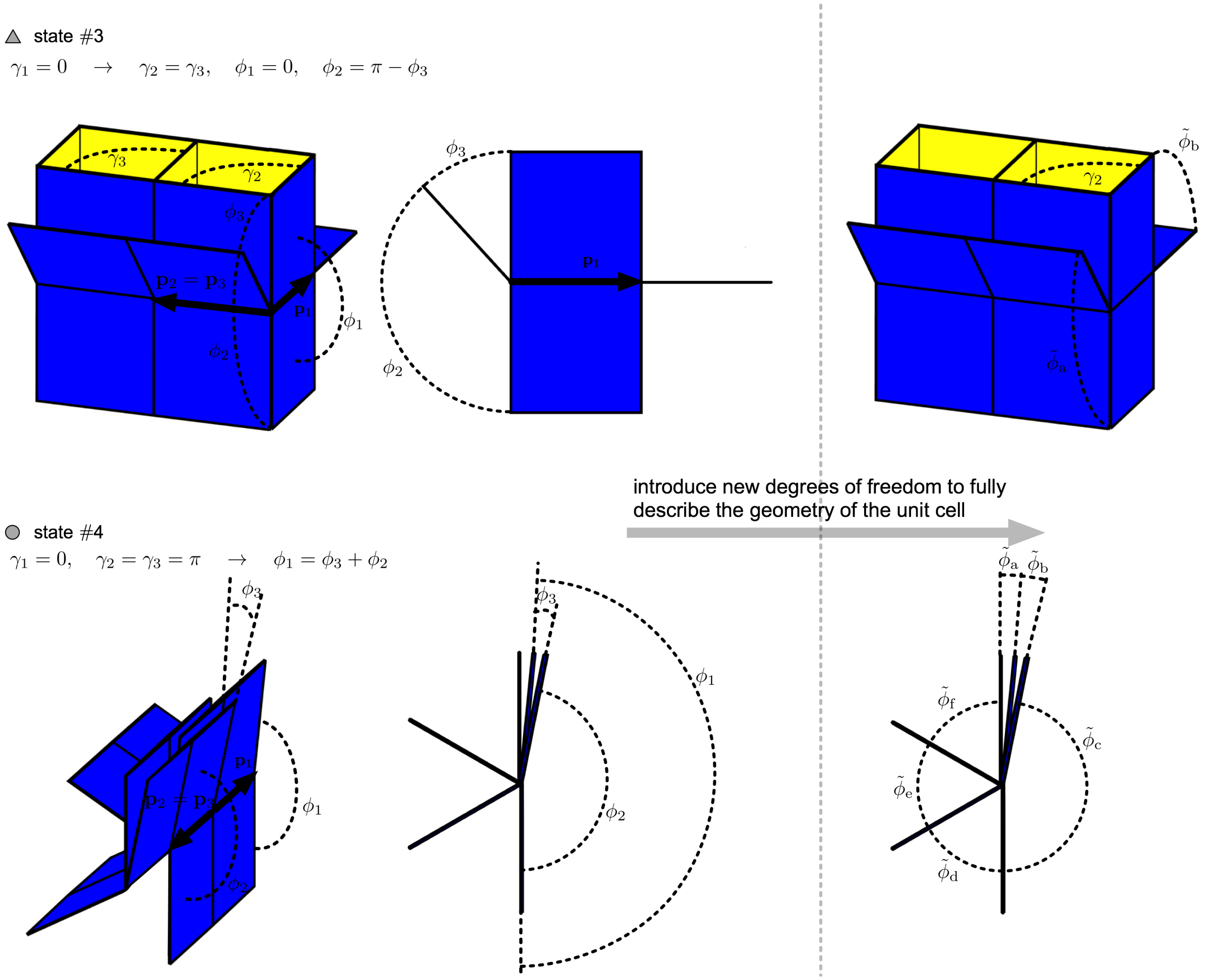

Supplementary Figure 1: Schematics indicating the γ and ϕ angles for the unit cell in state #3 (defined by $\gamma_1 = 0$ and $\gamma_2 = \gamma_3$) and state #4 (defined by $\gamma_1 = 0$ and $\gamma_2 = \gamma_3 = \pi$). For both states some of the rhombi flatten and their tilting angle cannot be described by the γ angles. As a result, a different set of degrees of freedom (indicated by the angles $\widetilde{\phi}$) need to be introduced to fully describe the geometry of the unit cell.

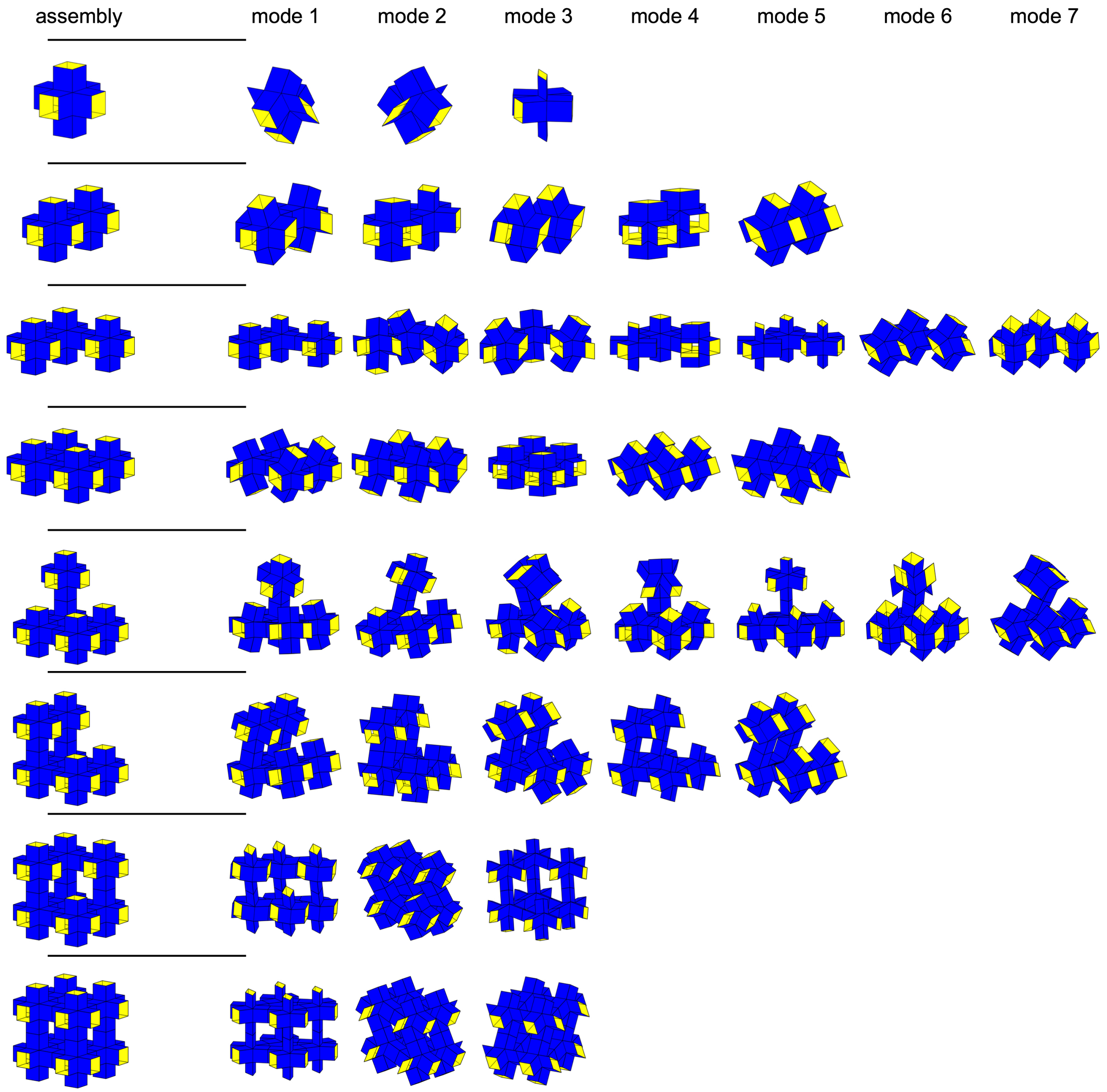

Supplementary Figure 2: Different assemblies and their corresponding folding modes. We have performed mode analysis on selected assemblies of different size using the commercial finite-element package Abaqus. We constructed 3D models of the assembly by modeling their faces as rigid plates (using coupling constraints between the nodes on a face in Abaqus) and the hinges as linear torsional springs (Abaqus hinge connector elements). We then determined all the natural frequency and corresponding modes for each assembly by performing a linear eigenfrequency analysis. Here, the number of modes directly correlates to the number of degrees of freedom, while the modes indicate the corresponding folding mechanism. This figure show eight different assemblies and their corresponding modes, as predicted by our numerical analysis. The results clearly show that both the number of unit cells and their connection affect the number of degrees of freedom. In particular, we find that when each unit cell has enough connections to neighboring cells (as for the 4×4×4 assembly) the total degrees of freedom of the assembly equals the number of degrees of freedom of the unit cell and the metamaterial deforms exactly in the same manner as the constituent unit cells. Differently, the number of degrees of freedom are found to increase for assemblies that have units cells with only a single connection to the other unit cells, or rows of unit cells that are only connected to the other unit cells on one side. In general, this analysis highlights that the design of metamaterials characterized by the same folding mechanisms of the constituent unit cells requires large enough connectivity.

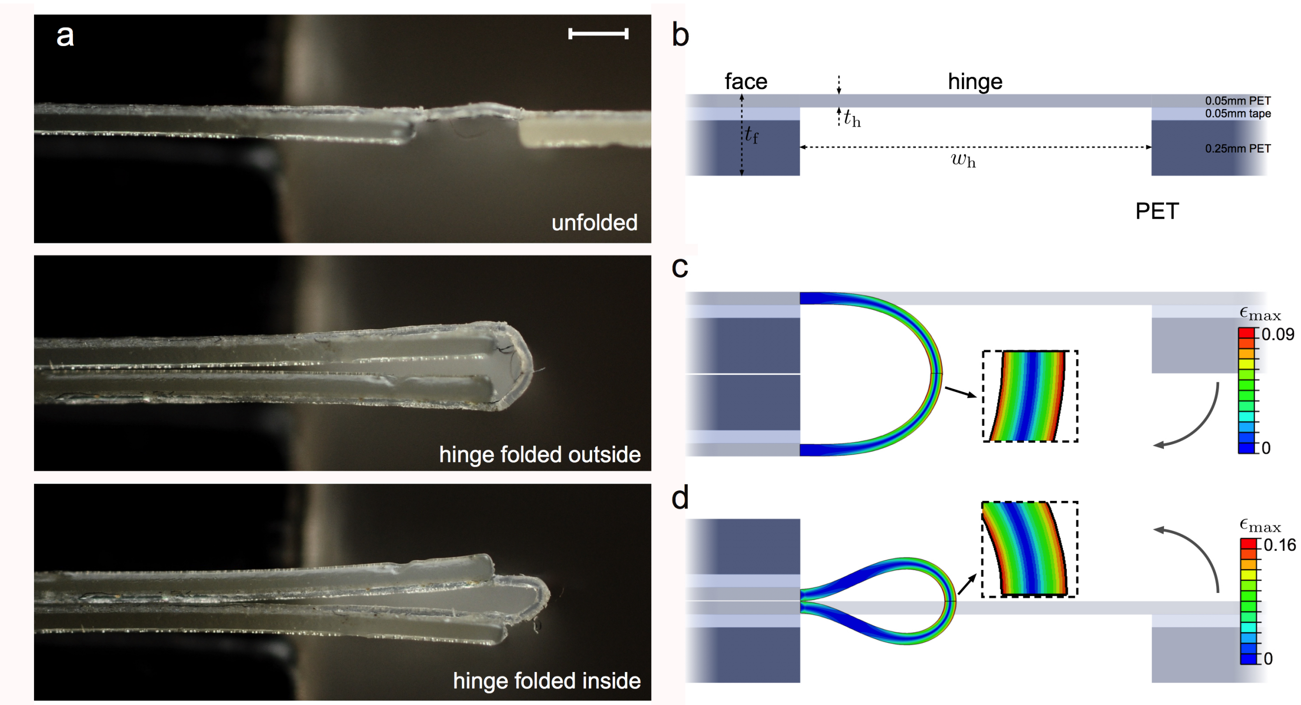

Supplementary Figure 3: (a) Close up of hinges in the undeformed and folded configuration (scale bar 1mm). (b) Schematic of the hinge. (c) and (d) show the results of finite element analysis conducted to predict the strains that occur in the hinges when completely folded to the outside and inside, respectively. In this set of simulations (conducted using the commercial software Abaqus) we considered a hinge of thickness $t_\mathrm{h} = 0.05$mm and width $w_\mathrm{h} = 1.5$mm connected on both ends to thicker sheets (the faces of the unit cell) with a thickness $t_\mathrm{f} = 0.35$mm. For the sake of simplicity, we modeled the faces as rigid bodies, while the stress-strain behavior of the hinges (which are meshed using plane strain quadrilateral quadratic elements, Abaqus element type CPE8R) was captured using a nearly incompressible Neo-Hookean material characterized by $K/\mu = 20$, where K is the bulk modulus and μ the shear modulus. The results of this analysis indicate that when the hinge is completely folded to the outside, the largest tensile and compressive strain equal $\epsilon_\mathrm{max} = 0.088$ and $\epsilon_\mathrm{min} = -0.091$. Larger strains ($\epsilon_\mathrm{max} = 0.164$ and $\epsilon_\mathrm{min} = -0.169$) are observed when the hinge is folded to the inside between the faces (Fig. **??**d). Therefore, when folding the unit cell completely flat, the hinges located on the inside of the faces will undergo strains that are 1.6 times larger then the elastic limit of approximately 10% [1]. Although the strains are larger than the elastic limit, they appear not to cause any permanent damage such as fracture or creases (sharp folds that are expected to form at $\epsilon_\mathrm{min} = -0.354$ [2]).

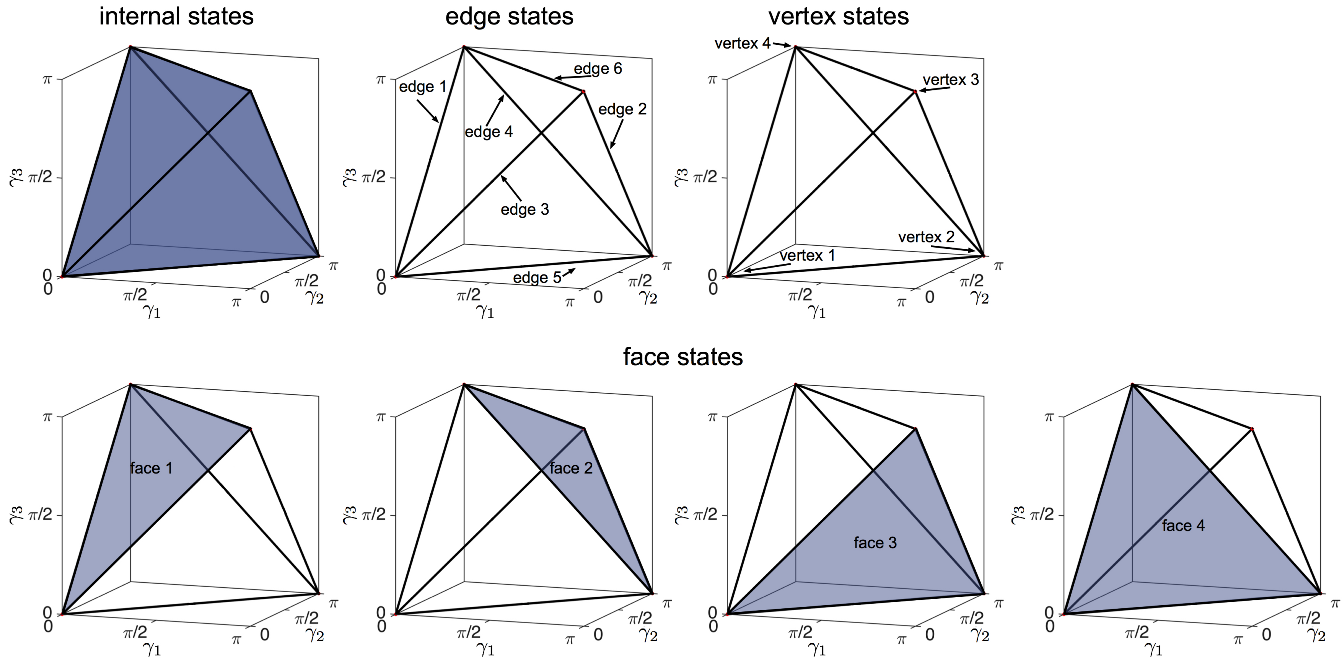

Supplementary Figure 4: Location of faces 1-4, edges 1-6 and vertices 1-4 as referred to in Supplementary Table 1.

Internal states				
$\gamma_3 < \gamma_1 + \gamma_2, \quad \gamma_3 < -(\gamma_1 + \gamma_2) + 2\pi, \quad \gamma_3 < (\gamma_1 - \gamma_2), \quad \gamma_3 \geq -(\gamma_1 - \gamma_2)$				
$\rightarrow \quad \phi_k = \arctan\left(\frac{\lvert(\boldsymbol{p}_j \times \boldsymbol{p}_k)\times(\boldsymbol{p}_k \times \boldsymbol{p}_i)\rvert}{(\boldsymbol{p}_j \times \boldsymbol{p}_k)\cdot(\boldsymbol{p}_k \times \boldsymbol{p}_i)}\right), \quad \text{for } (i,\, j,\, k) \in \{(1,\,2,\,3),\,(3,\,1,\,2),\,(2,\,3,\,1)\}$				
Face states				
face 1	$\gamma_3 = \gamma_1 + \gamma_2$	$\rightarrow \phi_1 = \phi_2 = 0, \quad \phi_3 = \pi$		
face 2	$\gamma_3 = -(\gamma_1 - \gamma_2) + 2\pi$	$\rightarrow \phi_1 = \phi_2 = \phi_3 = \pi$		
face 3	$\gamma_3 = \gamma_1 - \gamma_2$	$\rightarrow \phi_1 = \pi, \quad \phi_2 = \phi_3 = 0$		
face 4	$\gamma_3 = -(\gamma_1 - \gamma_2)$	$\rightarrow \phi_1 = \phi_3 = 0, \quad \phi_2 = \pi$		
Edge states				
edge 1	$\gamma_1 = 0, \quad \gamma_2 = \gamma_3$	$\rightarrow \boldsymbol{p}_2 = \boldsymbol{p}_3$	$\rightarrow \phi_1 = 0, \quad \phi_2 = \pi - \phi_3$	ϕ_2 and ϕ_3 indeterminate
edge 2	$\gamma_1 = \pi, \quad \gamma_2 = \pi - \gamma_3$	$\rightarrow \boldsymbol{p}_2 = -\boldsymbol{p}_3$	$\rightarrow \phi_1 = \pi, \quad \phi_2 = \phi_3$	ϕ_2 and ϕ_3 indeterminate
edge 3	$\gamma_2 = 0, \quad \gamma_1 = \gamma_3$	$\rightarrow \boldsymbol{p}_1 = \boldsymbol{p}_3$	$\rightarrow \phi_2 = 0, \quad \phi_1 = \pi - \phi_3$	ϕ_1 and ϕ_3 indeterminate
edge 4	$\gamma_2 = \pi, \quad \gamma_1 = \pi - \gamma_3$	$\rightarrow \boldsymbol{p}_1 = -\boldsymbol{p}_3$	$\rightarrow \phi_2 = \pi, \quad \phi_1 = \phi_3$	ϕ_1 and ϕ_3 indeterminate
edge 5	$\gamma_3 = 0, \quad \gamma_1 = \gamma_2$	$\rightarrow \boldsymbol{p}_1 = \boldsymbol{p}_2$	$\rightarrow \phi_3 = 0, \quad \phi_1 = \pi - \phi_2$	ϕ_1 and ϕ_2 indeterminate
edge 6	$\gamma_3 = \pi, \quad \gamma_1 = \pi - \gamma_2$	$\rightarrow \boldsymbol{p}_1 = -\boldsymbol{p}_2$	$\rightarrow \phi_3 = \pi, \quad \phi_1 = \phi_2$	ϕ_1 and ϕ_2 indeterminate
Vertex states				
vertex 1	$\gamma_1 = \gamma_2 = \gamma_3 = 0$	$\rightarrow \boldsymbol{p}_1 = \boldsymbol{p}_2 = \boldsymbol{p}_3$	$\rightarrow \phi_1 = 2\pi - \phi_2 - \phi_3$	ϕ_1, ϕ_2 and ϕ_3 indeterminate
vertex 2	$\gamma_1 = \gamma_2 = \pi, \quad \gamma_3 = 0$	$\rightarrow \boldsymbol{p}_1 = \boldsymbol{p}_2 = -\boldsymbol{p}_3$	$\rightarrow \phi_1 = -\phi_2 + \phi_3$	ϕ_1, ϕ_2 and ϕ_3 indeterminate
vertex 3	$\gamma_1 = \gamma_3 = \pi, \quad \gamma_2 = 0$	$\rightarrow \boldsymbol{p}_1 = -\boldsymbol{p}_2 = \boldsymbol{p}_3$	$\rightarrow \phi_1 = \phi_2 - \phi_3$	ϕ_1, ϕ_2 and ϕ_3 indeterminate
vertex 4	$\gamma_1 = 0, \quad \gamma_2 = \gamma_3 = \pi$	$\rightarrow \boldsymbol{p}_1 = -\boldsymbol{p}_2 = -\boldsymbol{p}_3$	$\rightarrow \phi_1 = \phi_2 + \phi_3$	ϕ_1, ϕ_2 and ϕ_3 indeterminate

Supplementary Table I: γ and ϕ angles defining the unit cell configurations corresponding to internal, face, edge and vertices states on the regular tetrahedron domain. Supplementary Figure 4 illustrates the location of faces 1-4, edges 1-6 and vertices 1-4.

SUPPLEMENTARY REFERENCES

[1] P. Colomban, Advances in Natural Sciences: Nanoscience and Nanotechnology **4**, 013001 (2013).
[2] E. Hohlfeld and L. Mahadevan, Physical Review Letters **106**, 105702 (2011).
